# A Condensation-Ordering Mechanism in Nanoparticle-Catalyzed Peptide Aggregation

**DOI:** 10.1371/journal.pcbi.1000458

**Published:** 2009-08-14

**Authors:** Stefan Auer, Antonio Trovato, Michele Vendruscolo

**Affiliations:** 1 Centre for Self Organising Molecular Systems, University of Leeds, Leeds, United Kingdom; 2 Dipartimento di Fisica, Universitá di Padova, CNISM and INFN, Padova, Italy; 3 Department of Chemistry, University of Cambridge, Cambridge, United Kingdom; National Institute of Diabetes and Digestive and Kidney Diseases, National Institutes of Health, United States of America

## Abstract

Nanoparticles introduced in living cells are capable of strongly promoting the aggregation of peptides and proteins. We use here molecular dynamics simulations to characterise in detail the process by which nanoparticle surfaces catalyse the self-assembly of peptides into fibrillar structures. The simulation of a system of hundreds of peptides over the millisecond timescale enables us to show that the mechanism of aggregation involves a first phase in which small structurally disordered oligomers assemble onto the nanoparticle and a second phase in which they evolve into highly ordered 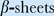 as their size increases.

## Author Summary

Protein misfolding and aggregation are associated with a wide variety of human disorders, which include Alzheimer's and Parkinson's diseases and late onset diabetes. It has been recently realised that the process of aggregation may be triggered by the presence of nanoparticles. We use here molecular dynamics simulations to characterise the molecular mechanism by which such nanoparticles are capable of enhancing the rate of formation of peptide aggregates. Our findings indicate that nanoparticle surfaces act as a catalyst that increases the local concentration of peptides, thus facilitating their subsequent assembly into stable fibrillar structures. The approach that we present, in addition to providing a description of the process of aggregation of peptides in the presence of nanoparticles, will enable the study of the mechanism of action of a variety of other potential aggregation-promoting agents present in living organisms, including lipid membranes and other cellular components.

## Introduction

With the advent of nanoscience much interest has arisen about the ways in which nanoparticles interact with biological systems, because of their potential applications in nanotechnology and effects on human health [1]–[7]. When nanoparticles are introduced in a living organism they may interact with a variety of different cellular components with yet largely unknown pathological consequences. These concerns have been articulated particularly in the case of misfolding disorders with increasing evidence, for example, about an association between exposure to heavy metals and an enhanced risk of developing Parkinson's disease [8]. Such misfolding diseases are caused by the aberrant association of peptides and proteins [9], which result in fibrillar aggregates that share a common 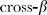 structure of intertwined layers of 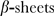 [9]. Although is well known that such aggregates are formed in a nucleation-dependent manner [9],[10] and that very often nucleation phenomena are known to be triggered by external factors [11], experimental reports on protein aggregation in heterogeneous systems have only begun to emerge [12]–[15]. These studies are important, since peptides and proteins *in vivo* often interact with a variety of potential seeding agents such as macromolecular complexes or membranes, which may strongly influence their aggregation behaviour. Indeed, it is well known that colloids [12],[14],[15], lipid bilayers [16], and liquid-air, liquid-solid or liquid-liquid interfaces [17],[18] can have significant effects in promoting amyloid formation. It has also been recently shown that, *in vivo*, nanoparticles are often covered by peptides and proteins that determine their behaviour in the cell [13],[15]. Despite these observations, the detailed processes underlying the association of proteins on surfaces or nanoparticles have so far remained elusive.

In this work we use molecular dynamics simulations to investigate the molecular mechanism of peptide self-assembly in the presence of spherical nanoparticles. Although computational studies using full atomistic models have provided considerable insight into the role of fundamental forces in promoting the self-assembly of polypeptide chains, they are restricted to relatively small systems of peptides and short timescales [19]–[29]. Coarse-grained models have proven capable of following the evolution of systems composed of larger numbers of peptides over longer timescales. The most tractable models are confined to a lattice [30]–[32], although in these cases the structural details used to represent polypeptide chain conformations are necessarily limited. Off-lattice protein models used to simulate protein aggregation include two-state models in which the protein can adopt, in addition to a native state, a state that is prone to 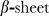 formation [33],[34], two-bead models, in which each amino acid is represented by two spheres with a knowledge-based potential [35], and fine-grained models with explicit representation of the side chains in combination with a phenomenological force field [36]–[39]. The more detailed is the protein model, the higher is the computational cost and the larger is the number of parameters required to specify the force field [40]. The studies mentioned above have investigated the process of protein self-assembly in homogeneous systems in which external factors such as nanoparticles or other molecules are absent. Only very recently, Friedman *et al.* investigated the process of assembly of amphiphatic peptides in the presence of lipid vescicles [41].

In the present work, we adopted an off-lattice protein model [42]–[45], in which the protein backbone is represented as tube that embeds a chain of 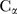 atoms subject to interactions that are common to all polypeptide chains, including excluded volume constraints, hydrophobic attractions, bending rigidity, and cooperative hydrogen bonds (see Materials and Methods). The major strength of the model is its ability to reproduce rather accurately secondary structure elements through the excluded-volume effects due to the tube geometry [42]–[45], which enables the use of a relatively simple force field and thus is very efficient computationally. By using this type of model, we already provided insight into the early stages of the aggregation process, to establish the existence of a general condensation-ordering transition for protein aggregation [46],[47], and to reveal a self-templated nucleation mechanism [48] that is able to explain a key feature observed in protein aggregation - the coupling between the initial formation of oligomeric assemblies and their subsequent rearrangement into a highly ordered 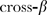 structures. In this work, we show the feasibility of simulating hundreds of peptides over several milliseconds, and we characterise in detail the molecular mechanism of self-assembly of the peptides at the surface of nanoparticles. This process takes place in two steps - at first the peptides associate on the surface thus increasing their local concentration and subsequently they undergo a process of reordering into 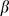 sheet structures, which is driven by the tendency to form hydrogen bonds.

## Results and Discussion

**Figure 1:**
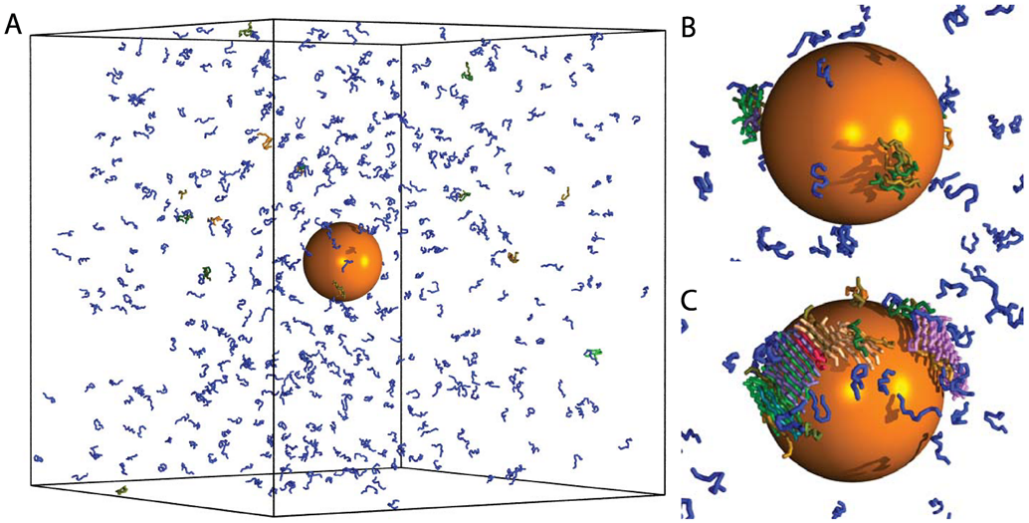
Illustration of the “condensation-ordering” mechanism of peptide self-assembly in the presence of a hydrophobic nanoparticle. (A) Initially, at 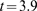 microseconds, the peptides are in their monomeric state. (B) At intermediate times, 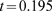 milliseconds, small oligomeric assemblies form on the nanoparticle surface. (C) At later times, 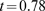 milliseconds, these oligomers re-order into fibrillar structures as their size increases. Peptides that do not form intermolecular hydrogen bonds are shown in blue, while peptides that form intermolecular hydrogen bonds are assigned a random colour, which is the same for peptides that belong to the same 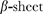. Two peptides are defined as belonging to the same cluster if their centres of mass distance is less than 5 Å. Two peptides are taken to participate within a 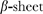 if they form more than four inter-chain hydrogen bonds with each other. The spherical nanoparticle is displayed in orange in the centre of the simulation box; the diameter of the peptides is slightly reduced for illustration purposes. Panels (B) and (C) show enlarged views of the nanoparticle-peptide system. The simulation was performed at 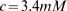, 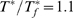, 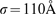, and 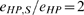.

Starting from the experimental observation that amyloid formation is a phenomenon common to most polypeptide chains [9], and that systems of polyamino acids have been shown to form amyloid assemblies [49],[50], we investigate the aggregation behaviour of 512 12-residue polyamino acids in the presence of spherical nanoparticles (Fig. 1A) as a model system to reveal the general properties of this phenomenon. The peptides that we considered exhibit an 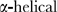 native structure below their folding temperature 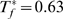 (expressed here in reduced units, see Materials and Methods) and an extended random coil structure above it. The aggregation behaviour that we observe depends on the diameter 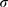 of the spherical nanoparticle and on the strength of the peptide-nanoparticle interaction, 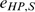, which is the energy gained when the distance between a 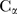 atom representing the peptide molecules and the nanoparticle surface is smaller than 10 Å. This range was chosen to be similar to that of the pairwise hydrophobic inter-residue interaction, since both interactions are effectively due to hydrophobic solvation effects.

We first performed molecular dynamics simulations at a peptide concentration, 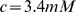, and reduced temperature, 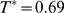, at which in absence of the nanoparticle aggregation does not occur on the timescale accessible to the simulation. At this temperature most peptides are unfolded since 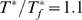. The nanoparticle diameter is set to 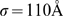 and the interaction strength between the nanoparticle and the peptides, 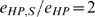, is set to twice the value of the hydrophobic attraction, 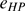, between different 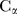 atoms. The results of a representative molecular dynamics trajectory in presence of this weakly hydrophobic nanoparticle are shown in Fig. 1.

**Figure 2:**
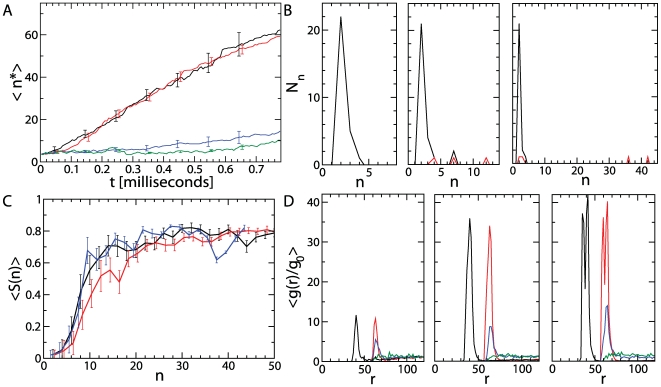
Structural analysis of the nanoparticle-induced self-assembly mechanism. (A) Average size of the largest cluster 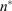 observed during a simulation in presence of a hard sphere nanoparticle: 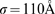, 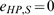 (green line), and several hydrophobic nanoparticles that differ in diameter and hydrophobicity: 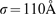, 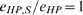 (blue line), 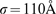, 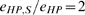 (red line), and 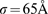, 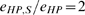 (black line). The results are averaged over ten independent simulation runs and the error bars correspond to the standard deviation of the mean. (B) Number of clusters 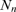 of size 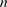 as a function of time: 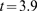 microseconds (left), 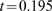 milliseconds (middle), 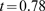 milliseconds (right), for the MD trajectory and parameters described in Fig. 1. Black lines correspond to all clusters formed in the system; red lines correspond to the number of clusters formed on the nanoparticle surface. (C) Structural order parameter 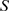 as a function of the cluster size 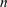 averaged over ten independent simulations. The line colours are as described in (A). (D) Normalized density profile 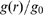, where 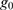 is the bulk density of the system, as a function of the distance from the centre of mass of the nanoparticle at the beginning of the simulation, 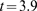 microseconds (left panel), intermediate times, 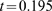 milliseconds (middle panel), and at the end 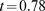 milliseconds (right panel). The different line colours are as described in (A) and correspond to the different seed sizes and peptide seed interaction energies. The results are averaged over ten independent simulations and the error bars correspond to the standard deviation of the mean.

We consistently observe that the presence of this hydrophobic nanoparticle effectively removes the lag-time prior to aggregation (Fig. 2A, red line) by triggering the condensation of peptides on the nanoparticle surface to initially form small disordered oligomers (Fig. 1B), which re-order into 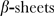 as their size increases (Fig. 1C). Although dimers and trimers constantly form and dissolve throughout the simulation (Fig. 2B), larger oligomers appear only on the nanoparticle surface and at later times. For example, at 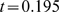 milliseconds, we observed one cluster of size 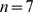 in solution, and two clusters of sizes 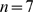 and 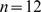 on the nanoparticle surface (see Figs. 1B, 2B, middle panel). At the end of the simulation, at 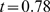 milliseconds, the two oligomers on the nanoparticle surface had grown to sizes 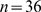 and 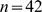, whereas the oligomers in solution dissolved (see Fig. 2B, right panel). Animations representing the molecular dynamics trajectory corresponding to Fig. 1 (Videos S1 and S2, Supplementary Material) also illustrate that the small oligomers on the seed surface can diffuse rather freely, and that two of them collide and merge into a larger one.

In order to provide a detailed analysis of the structure of the clusters that form on the nanoparticle surface we calculated the liquid crystalline order parameter 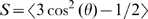, a measure for the alignment between different strands in a single 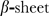 and between different 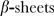, where 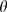 is the angle between neighbouring peptides within an aggregating cluster. Our calculations confirm that small clusters are disordered and only larger ones reach a value 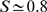 characteristic for liquid crystalline ordering (Fig. 2C). To investigate the effect of the nanoparticle on the local structure around its surface we calculated the density profile of peptides as a function of their distance from the centre of mass of the nanoparticle. Our results illustrate that the presence of a hydrophobic nanoparticle leads to the formation of a high density shell at the nanoparticle surface, which becomes more pronounced as the simulation progresses (Fig. 2D). The enhanced density of peptides at the nanoparticle surface increases the probability to form clusters, which will eventually trigger the formation of small clusters on the seed surface. The appearance of a double layer structure seems to be dependent on the reaching of a local density threshold (compare different curves in 2D, rightmost panel). The facilitation of the nucleation step within an intermediate dense assembly is well known in crystallisation [51] and was also observed in the assembly of peptides into 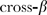 structures [47]. These results also provide a molecular illustration of the dynamics of polypeptide chains associated with the “corona” effect observed in recent experiments [13],[15], which has revealed that *in vivo* nanoparticles are always covered by biological molecules.

In our simulations the lag time for nanoparticle induced peptide aggregation is about a microsecond which is quite short compared to the lag times typically observed in experiments. The latter range from some hours to several days, but it should be noted that both peptide concentration (3.4 mM) and, especially, nanoparticle concentration (6.5 µM) are much higher than in experiments (40–80 µM and 4–90 pM, respectively) [12]. This implies that in our simulations nucleation barriers are essentially removed by the nanoparticle whereas in experiments they are still high.

**Figure 3:**
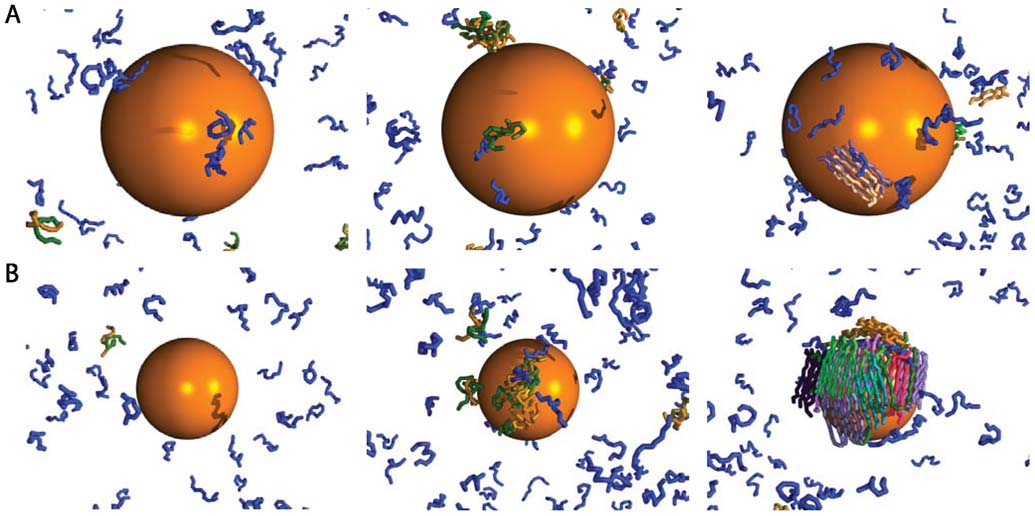
Illustration of the condensation-ordering mechanism for different hydrophobicity of the nanoparticle, nanoparticle diameter. (A) 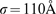, 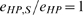 at 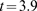 microseconds(left), 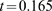 milliseconds (middle), 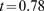 milliseconds (right). (B) 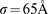, 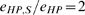 at 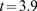 microseconds (left), 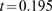 milliseconds (middle), 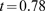 milliseconds (right). The concentration and temperature are 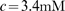, 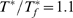 respectively, and the colour code is as described in Fig 1.

In our model system the binding of peptides to the nanoparticle is stronger for the more hydrophobic surface (Fig. 2D). As a result, increasing the hydrophobicity of the nanoparticle reduces the lag time prior to aggregation (Figs. 2A and 3). The same correlation between a stronger nanoparticle-protein binding and a more enhanced reduction of the lag time prior to aggregation is found in experiments on 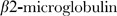 fibrillation in the presence of hydrophobic copolymer nanoparticles [12]. This supports the already suggested notion that a hydrophobic nanoparticle favors aggregation by leading to a local increase in peptide concentration around its surface. Note that, as 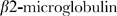 is found to bind more weakly to the more hydrophobic nanoparticles, the latter are found to be *less* effective as well in reducing aggregation lag times [12]. Both these facts are not reproduced in our simulations, reflecting our most simple modeling of the hydrophobic effect and of the internal structure of both the nanoparticle and the protein.

We did not observe an increase of the lag time prior to aggregation by using a smaller nanoparticle diameter, 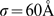. The fluctuations in the size of the largest cluster are nevertheless larger, indicating that the bigger nanoparticle is a slightly more efficient seed. Experimentally, it was shown that curvature effects can strongly affect the fibrillation kinetics in a way which depends on solution conditions [12]. In our simulations we simply cannot observe this effect because the nucleation barriers are effectively removed by the nanoparticle.

As a final remark, we observe that the molecular mechanism associated with the condensation ordering transition for peptide nanoparticle association described here is independent of particle size and hydrophobicity. The structural reorganization of protein chains in the early disordered oligomeric assemblies from their native or unstructured conformation to the 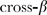 state may be more easily observed by experiments using a nanoparticle as it localizes the nucleation event, which may enable to monitor the reorganization process by fluorescence methods.

### Conclusions

We have characterised the process of nanoparticle-catalysed peptide aggregation in terms of a condensation-ordering mechanism and investigated its dependence on the nanoparticle diameter and the strength of the nanoparticle-peptide interactions. A similar mechanism of aggregation has already been observed in the absence of catalysing factors [46]–[48],[52], suggesting that the process of aggregation is driven in both cases by the intrinsic tendency of polypeptide chains to associate by forming ordered networks of hydrogen bonds [53],[54]. In the case that we have studied here, the initial condensation of peptides is initiated by nanoparticle surfaces to form small disordered oligomeric structures that subsequently re-order into 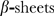 as their size increases. Although this mechanism will be modulated by specific sequence-dependent interactions for more complex amino acid sequences, our findings are consistent with recent experiments on seeded fibrillation [12]. These results therefore suggest that the process of protein aggregation can be speeded up by the presence of factors capable of increasing the local concentration of proteins and thus promoting the formation of disorder oligomeric assemblies whose presence in turn facilitates the conversion of soluble proteins into highly ordered fibrillar structures.

## Materials and Methods

### Description of the model

We used a modified version of the tube model [42]. In this model, residues are represented by their 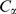 atoms, which are connected into a chain with a distance of 3.8±0.2 Å between neighbouring atoms. The tube geometry is approximated by assigning a diameter of 3.8 Å to the 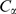 atoms. Neighbouring 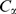 atoms are not allowed to interpenetrate. Bond angles are restricted between 82° to 148°, and an analogue of bending rigidity is introduced by means of an energetic penalty, 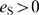, for values of bond angles lower than 107.15°; these are the same criteria used previously [42]. The introduction of 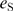 is useful to mimic the constraints placed on local conformations by the presence of side chains, as usually visualised by Ramachandran plot. Hydrophobicity enters through a pairwise-additive interaction energy of 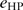 (positive or negative) between any pair of residues 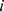 and 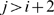 that approach closer than 7.5 Å.

**Figure 4:**
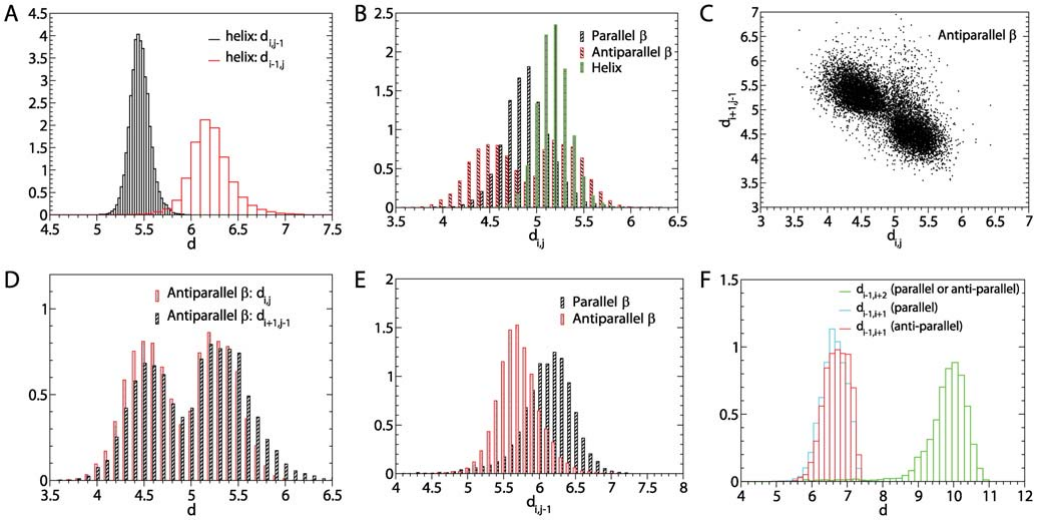
Normalised histograms of distances between 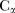 atoms involved in backbone-backbone hydrogen bonds. The analysis is based on 500 high resolution PDB structures [55] and used to define hydrogen bonds in the protein model employed in our simulation. (A) Histogram of distances 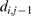 and 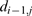 between 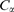 atoms 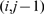 and 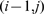 used to define a 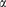 helical hydrogen bond assigned to atoms 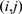 with 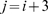. (B) Histogram of distances 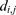 between 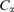 atoms 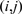 that form a parallel, anti-parallel, or helical hydrogen bond. For the 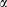 helical hydrogen bond 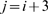. (C) Illustration of the alternation of distances for consecutive 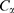 atoms that form anti-parallel hydrogen bonds. (D) as in (C). (E) The distance 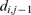 is used to define hydrogen bonds between atoms 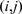 in parallel and anti parallel 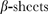. (F) Distances used to define cooperative hydrogen bonds between two consecutive atoms 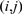 and 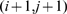 that form parallel 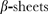 or 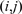 and 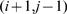 that form anti parallel 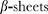.

The quasi-cylindrical symmetry of the tube is broken by the geometric requirements of hydrogen bonds. These geometrical requirements were deduced from an analysis of 500 high resolution PDB native structures [55], from which we computed the normalised histograms of distances between 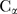 atoms involved in backbone-backbone hydrogen bonds which are shown in Fig. 4. The distances we used to define the hydrogen bonds at the 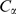 atom level are summarised in Table 1. Our definitions distinguish between hydrogen bonds that belong to a 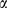 helix, parallel or anti-parallel 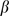 sheets. We emphasise the fact that there is not a full correspondence with the real hydrogen bonds formed between amide and carboxyl backbone groups. For instance, there are two different kinds of residue pairs facing each other in nearby anti-parallel 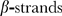. In the first kind, the two hydrogen bonds are formed between the two residues, whereas in the second kind, no hydrogen bond is formed between them. The two kinds alternate along the pair of nearby strands. In our definition of hydrogen bonds based on 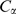 atoms, we will say that for both kind of pairs one hydrogen bond is formed between the two 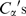. Yet, we keep track of the peculiar geometry of hydrogen bonds within anti-parallel 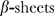 by using two different sets of distances, which we call anti-parallel 1 and anti-parallel 2, as the distances between consecutive 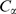 pairs facing each other on nearby 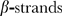 do indeed alternate. Furthermore, we request that one residue cannot form more than two hydrogen bonds, and that the first and last 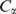 atoms of a peptide do not at all. Hydrogen bonds may form cooperatively between residues 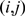 and 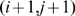 [or 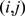 and 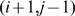 for anti-parallel hydrogen bonds], thereby gaining an additional energy of 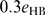. The distance criteria for cooperative hydrogen bonds within 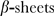 are obtained from Fig. 4F and summarised in Table 1.

**Table 1:** Summary of distances used to define the various hydrogen bonds.

α helix			β (parallel)			β (anti-parallel 1)			β (anti-parallel 2)			co-operativity		
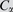	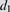	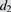	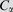	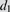	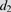	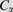	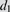	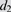	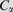	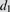	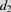	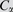	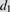	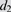
(i,i+3)	4.75	5.6	(i,j)	4.3	5.5	(i,j)	3.95	4.8	(i,j)	4.9	5.7	(i−1,i+1)	5.6	7.4
(i,i+2)	5.1	5.7	(i,j−1)	5.3	6.85	(i,j−1)	4.95	6.25	(i,j−1)	5.05	6.3	(i,i+2)	5.65	7.5
(i+1,i+3)	5.2	5.75	(i−1,j)	5.2	6.95	(i−1,j)	5.0	6.4	(i−1,j)	5.1	6.5	(i−1,i+2)	9.5	10.8
(i−1,i+3)	5.8	6.7	(i+1,j+1)	4.1	5.6	(i+1,j−1)	4.7	6.0	(i+1,j−1)	3.8	5.25	(j−1,j+1)	5.6	7.4
			(i−1,j−1)	4.2	5.65	(i−1,j+1)	4.75	6.1	(i−1,j+1)	3.85	5.35	(j,j+2)	5.65	7.5
			(i+1,j−1)	6.15	9.9							(j−1,j+2)	9.5	10.8

Here 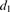 and 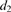 are the lower and the upper threshold distances obtained from Fig. 4 or from similar histograms. The hydrogen bonds are formally assigned to 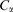 atoms 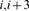 (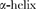), 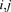 (parallel and anti-parallel 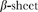), and the cooperative hydrogen bonds are formed between the pairs 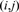 and 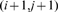 for parallel 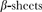 or between the pairs 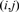 and 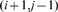 for anti-parallel 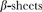.

The energy of hydrogen bonds was set to 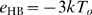, where 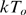 is the thermal energy at room temperature and 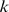 is the Boltzmann constant. This energy correspond to the experimental one (1.5 kCal/mol at room temperature [56]). Values of the hydrophobicity and stiffness parameters, 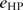 and 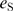, are given in units of 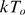 and the reduced temperature is 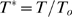. In all our simulations we set 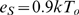 and 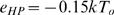. The ratio of a hydrogen bonding energy to hydrophobic energy is 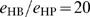. As the number of hydrophobic contacts within an oligomer is usually about one order of magnitude larger than the number of hydrogen bonds, our choice ensures that these interactions provide similar contributions to the potential energy of the oligomer [47]. For this set of model parameter the peptide folds into a native 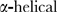 state below the folding temperature 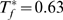. 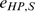 is the parameter which determines the strength of the interaction energy between 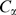 atoms representing the peptide molecules and the seed particle. The range of the peptide seed interaction is set to 10 Å from the nanoparticle surface.

### Simulation techniques

We performed discontinuous molecular dynamics (DMD) simulations [57], which is a fast alternative to standard molecular dynamics simulations. The main difference is that in DMD simulations the system evolves on a collision by collision basis, and requires the calculation of the collision dynamics and the search for the next collision. In the simulations we used a cubic box, of side 633 Å, and applied periodic boundary conditions. The implementation of our definition for the hydrogen bonding requires some additional consideration. In order to prevent that one residue forms three hydrogen bonds we treat the associated collision as fully elastic. In order to implement and consider cooperative hydrogen bonding we keep and update a list of all hydrogen bonds formed in the system at all times. Note that a recalculation of the hydrogen bonds formed in the system without considering this list can lead to a different result. Independent starting configurations were generated at 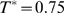 and rapidly cooled down to 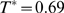 at the beginning of each simulation run. We performed all our simulation in the NVT ensemble using an Anderson thermostat.

In order to associate the number of collision steps performed in our simulation to a real time we measured the long time self-diffusion coefficient of our model peptide, 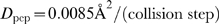, and matched it to experimental data. We took from the literature the value for the self-diffusion coefficient, 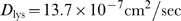, which was measured for lysozyme [43]. The Einstein relation for the diffusion coefficient together with the Stokes law yield 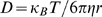 where 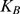 is the Boltzmann constant, 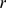 is the radius of the diffusing object, and 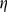 is the viscosity. The latter can be evaluated through kinetic theory as 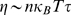, where 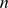 is the density of the viscous medium in which diffusion takes place and 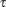 is the mean flight time between collision with solvent molecules setting the time scale [15]. The resulting expression for the diffusion coefficient 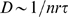 allows us to get 
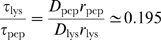
 picoseconds as an estimate of the real time corresponding to one collision step in our molecular dynamics simulations. We use 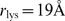 as an estimate of 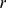 for lysozyme, whereas we take 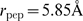 as the average radius of gyration of the peptide as found in our simulations. Hence, the total number of collision steps, 4×10^9^, performed in every simulation corresponds qualitatively to 0.78 milliseconds.

## Supporting Information

Video S1 Configurations obtained from the molecular dynamics trajectory that corresponds to Fig. 1.
(5.47 MB GIF)

Video S2 Final configuration obtained from the molecular dynamics trajectory shown to Fig. 1.
(3.25 MB GIF)
