# Supplementary figures and images for: A Condensation-Ordering Mechanism in Nanoparticle-Catalyzed Peptide Aggregation
Source: PLoS Comput Biol. 2009 Aug 14;5(8):e1000458. doi: 10.1371/journal.pcbi.1000458 (PMC2715216; doi:10.1371/journal.pcbi.1000458)

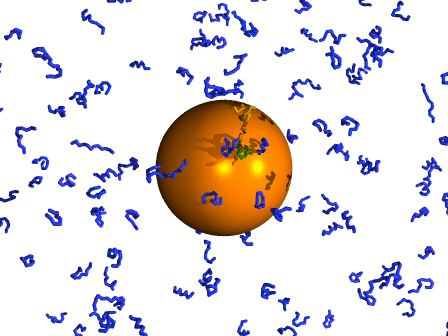

Supplement: Video S1 — Configurations obtained from the molecular dynamics trajectory that corresponds to Fig. 1. (5.47 MB GIF) [file pcbi.1000458.s001.gif]

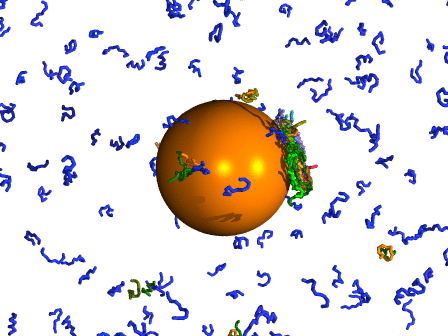

Supplement: Video S2 — Final configuration obtained from the molecular dynamics trajectory shown to Fig. 1. (3.25 MB GIF) [file pcbi.1000458.s002.gif]
